# Thermal and Sound Insulation Properties of Organic Biocomposite Mixtures

**DOI:** 10.3390/polym16050672

**Published:** 2024-03-01

**Authors:** Mihai Alin Pop, Cătălin Croitoru, Simona Matei, Sebastian-Marian Zaharia, Mihaela Coșniță, Cosmin Spîrchez

**Affiliations:** 1 Department of Materials Science, Transilvania University of Brasov, 500036 Brasov, Romania; mihai.pop@unitbv.ro (M.A.P.); simona.matei@unitbv.ro (S.M.); 2 Materials Engineering and Welding Department, Transilvania University of Brasov, 500036 Brasov, Romania; 3 Department of Manufacturing Engineering, Transilvania University of Brasov, 500036 Brasov, Romania; zaharia_sebastian@unitbv.ro; 4 Department of Product Design, Mechatronics and Environment, Transilvania University of Brasov, 500036 Brasov, Romania; mihaela.cosnita@unitbv.ro; 5 Wood Processing and Design Wooden Product Department, Transilvania University of Brasov, 500036 Brasov, Romania; cosmin.spirchez@unitbv.ro

**Keywords:** thermal properties, sound insulation, biocomposites, paper pulp, internal architectures

## Abstract

Sustainable building materials with excellent thermal stability and sound insulation are crucial for eco-friendly construction. This study investigates biocomposites made from cellulose pulp reinforced with beeswax, fir resin, and natural fillers like horsetail, rice flour, and fir needles. Eight formulations were obtained, and their thermal resistance, oxidation temperature, and acoustic properties were evaluated. Biocomposites exhibited significant improvements compared to conventional materials. Oxidation temperature onset increased by 60–70 °C compared to polyurethane foam or recycled textiles, reaching 280–290 °C. Sound absorption coefficients ranged from 0.15 to 0.78, with some formulations exceeding 0.5 across mid-frequencies, indicating good sound-dampening potential. These findings demonstrate the promise of these biocomposites for sustainable construction, offering a balance of thermal and acoustic performance alongside environmental and health benefits.

## 1. Introduction

A balance between energy, performance, and cost efficiency are critical issues in all engineering fields. Nearly 40% of energy consumption and 36% of greenhouse gas emissions are caused by residential and commercial buildings. Therefore, there are initiatives to reduce these emissions and energy consumption while increasing the sustainability of the built environment through the concept of near-zero energy (NZE) buildings [1]. One of the key aspects of NZE buildings is the use of sustainable and energy-efficient materials for construction. In response to this challenge, one ongoing research direction is aimed at developing new eco-friendly, non-structural multipurpose materials with sound- or thermal-insulating properties [2]. In this context, all-natural composite materials have emerged as a promising alternative to synthetic polymer composites for various applications, including sound insulation and non-structural panels. 

This paper focuses on the thermal stability and sound insulation properties of all-natural composites, as these are crucial factors in determining their suitability for use in various applications, such as building insulation, acoustic panels, packaging, and furniture. Thermal stability is crucial for ensuring the long-term performance and durability of the materials used in construction, while sound insulation is vital for maintaining a comfortable and quiet living environment. By understanding the thermal and acoustic properties of all-natural composites, their performance can be optimized and sustainable, and alternatives can be designed.

The benefits of using ecological materials instead of synthetic ones as insulation include lower energy and carbon usage, as well as esthetic perks like unique textures, colors, and patterns [3,4]. These ecological materials could also promote a healthier indoor environment, including the elimination of volatile organic compounds [5,6,7,8]. In some cases, it has been found that, for example, natural composites have lower thermal conductivity and higher thermal diffusivity than synthetic composites, which means they can better resist heat transfer and thermal shocks [7]. Also, natural composites can have higher sound absorption coefficients than synthetic composites, which means they can better absorb and dissipate sound energy [8]. 

Natural formulations, such as paper, natural fibers, waxes, and natural resins, have been widely used as components of composite materials due to their availability, cost, renewability, biodegradability, and compatibility with each other. However, the sound performance and thermal properties of hybrid all-organic composites with natural resources as both matrix and filler have not been extensively studied. 

Therefore, this work aims to assess the evaluation of the thermal properties (first-order transitions, glass transitions, degradation, and stability against cyclic thermal aging) and acoustic properties (sound absorption coefficient—SAC, sound transmission loss—STL) of several tailor-made all-organic composites from natural resources. The composites present an organic matrix (beeswax, fir resin) and biomass fillers (horsetail powder, rice flour, ground fir needles, recycled cellulose fibers from paper) in varying amounts. In this assembly, paper and fibers can provide structural reinforcement and thermal insulation, while wax and natural resins can act as binders and modifiers of the composite properties [9]. 

The advantage of using beeswax and fir resin as the matrix could be their improved compatibility with the filler, as they contain a wide range of compounds in their structure, including hydrocarbons with a chain length of C27-C33, free fatty acids with a chain length of C24-C32, and fatty acid esters in the case of beeswax [10] and terpenes and rosin acids in the case of fir resin [10], as well as other compounds. Also, among the advantages, the easy processing may be mentioned (due to their relatively lower melting/softening points compared to synthetic thermoplastics, for example). According to Neto et al. [9], the thermal stability of beeswax and its composites with different fillers was investigated by TGA and DSC. The results showed that beeswax had a melting point of about 65 °C and a degradation temperature of about 300 °C. The addition of fillers, such as wood flour or rice husk, increased the thermal stability of the composites by delaying the onset of degradation and reducing weight loss [11]. Beeswax can be used as a matrix or a modifier in composites with different fillers, such as wood, rice husk, calcium carbonate, glass fiber, carbon fiber, and clay. Beeswax composites showed good thermal, mechanical, and electrical properties, but their sound absorption properties have not been widely studied [12]. 

According to [13], the thermal stability of coniferous resin and its composites with different fillers was studied by TGA and DSC. The results showed that fir resin had a softening point of about 80 °C and a degradation temperature of about 350 °C, which means that it can withstand moderate heating without significant changes to its properties [14]. The melting point depends on the type and purity of the resin and can vary from 60 to 120 °C. Coniferous resins can be used as matrices or modifiers in composites with different fillers, such as wood, cellulose, glass fiber, carbon fiber, and clay, which generally improve thermal stability by increasing the onset of degradation and decreasing weight loss. Coniferous resin composites also showed good mechanical and electrical properties, but their sound absorption properties have not been reported [15]. 

Natural fibers and ground plant material, in general, and their polymer composites with synthetic matrices (polyesters, thermosets) have different thermal degradation temperatures depending on their chemical composition, morphology, molecular structure, and interfacial interactions. Generally, cellulose degrades at around 300 °C, hemicellulose degrades at around 200 °C, and lignin degrades at around 400 °C [16]. The addition of plant fibers, bacterial cellulose fibers, or ground plant-based material to thermoplastic matrices, such as polypropylene, polyethylene, and polylactic acid, enhanced the thermal stability of the composites by increasing the char residue and decreasing the weight loss [17]. Biomass-derived fillers can influence the thermal conductivity, thermal expansion, and thermal degradation of the natural composites by altering the heat transfer and thermal stability of the matrix. The thermal properties depend on the thermal conductivity, specific heat, and thermal degradation temperature of the fillers, as well as the thermal contact resistance and thermal mismatch between the fillers and the matrix [18].

Horsetail and fir needles used in this study contain, in addition to the lignocellulosic part, phenolic compounds, resin acids, or silicon dioxide (horsetail), which have a strengthening and thermal stabilizing effect on wax and/or resin. Rice flour contains starch, waxes, and proteins that are compatible with the chosen matrices, and ground paper (recycled cellulose) has a high specific surface area and a high degree of absorption/wicking for wax and resin. 

Fiber and paper composites have been extensively studied for their sound absorption properties. Various factors, such as composite structure, fiber-based thickness, and surface treatment, were investigated to optimize the sound absorption coefficient [19]. Generally, natural plant-derived fillers have lower density and higher porosity than synthetic fillers, which is beneficial for sound insulation applications. Also, natural plant-derived fillers have more irregular shapes than synthetic fillers, which can increase the overall irregularity of the composites, helping with sound wave scattering, diffraction, or reflection [20,21,22].

Chen et al. developed multi-layer gradient fiber-based composites with porous, resonant, and damping structures, achieving a broadband sound absorption coefficient (SAC) of 0.42 [23]. Khem et al. studied the sound absorption capability of oil palm frond (OPF) composites and found that the sound absorption coefficient was proportional to density and improved with alkali treatment [24]. Haghighat et al. evaluated the impact of fiber size on the sound absorption values of sugarcane bagasse (SCB) waste fiber composites and found that smaller fiber sizes resulted in higher absorption performance [25]. The sound absorbing coefficient for paper pulp composites can vary between 0.2 and 0.6, depending on factors such as fiber density and composition [26]. 

This study explores the synergy between natural components (beeswax, fir resin, rice flour, and horsetail) and recycled paper pulp to create fully bio-based composites with superior thermal stability and sound insulation. While previous research examined these components individually, their combined acoustic insulation potential remains largely unexplored. This study addresses this issue by evaluating how formulation adjustments and internal composite geometry (patterned voids) affect sound absorption, transmission, and reflection characteristics.

Compared to synthetic polymer composites, these fully bio-based materials could offer significant environmental and economic advantages, including lower environmental impact and, potentially, lower cost. However, a comprehensive understanding of their sound absorption and thermal degradation properties is lacking. This study aims to fill this knowledge gap by investigating the influence of composition, structure, and natural component properties on composite performance, ultimately paving the way for optimized materials for diverse applications.

## 2. Experimental

### 2.1. Materials

The natural beeswax was of the type of artificial combs used in beekeeping. The horsetail (*Equisetum arvense*) plant, rice, fir resin, and fir needles were purchased from a store specializing in natural products (Paradisul Verde, Brasov, Romania). The recycled paper used was obtained by grinding waste document fragments (usual A4 paper with a specific weight of 80 g/m^2^).

### 2.2. Mixtures Obtaining

A total of 8 materials recipes were obtained by melting beeswax and fir resin and mixing it with horsetail/fir needles and ground powder/rice flour, respectively, in predetermined proportions and pouring it into circular silicone rubber molds, resulting in specimens with a diameter of 18 mm and a height between 15 and 20 mm. The composite recipes expressed in Table 1 represent the composition of wax, resin, and ground biomass fillers that were poured over the cellulose fiber pulp base to obtain the sound insulation composites, as detailed in Section 2.5.

The recipes mentioned in Table 1 were chosen based on our preliminary experimentation. The filler integration and spreading behavior of each component (fir resin, beeswax, and their mixture) was evaluated with increasing filler(s) content. This iterative process aimed to identify the maximum filler incorporation possible while maintaining good processability and uniform spreading on the paper pulp core.

The miscibility of hydrophilic and hydrophobic materials at the filler–matrix interface is crucial in controlling dispersion and determining the final composite properties. Poor compatibility could lead to phase separation or weak interfacial bonding. 

Several factors help mediate the hydrophilicity discrepancy in our formulations. First, waxes and resins contain ester, acid, and alcohol groups, making them moderately polar and capable of hydrogen bonding with cellulose. This increases interfacial interaction. Secondly, fillers like horsetail and rice contain various polar extractives and surface-active compounds that can plasticize the interface. Heating the mixtures above the melting points enhances molecular mobility and mixing, and strong mechanical processes like grinding disrupt filler particle agglomeration, increasing surface area for interaction.

### 2.3. Thermal Analysis

The thermal analysis (TA) of the compositions corresponding to Table 1 was conducted using a Differential Scanning Calorimetry (DSC) technique with a DSC-200 F3 Maia/STA 449F3 Jupiter (Netzsch, Selb, Germany) instrument. The thermal protocol consisted of a heating and cooling rate of 10 °C/min, with the following steps: (1) cooling from room temperature (25 °C) to −80 °C; (2) heating from −80 °C to 300 °C; (3) cooling from 300 °C back to room temperature.

The composite samples underwent cyclic thermal aging by being subjected to five consecutive cooling/heating cycles between −70 °C and 70 °C using the same DSC instrument and 10 °C/min heating/cooling rate as previously described.

The simultaneous thermal analysis (STA) combines Thermogravimetry (TG) and Differential Scanning Calorimetry (DSC) into a single instrument. The sample analysis was conducted in a temperature range of 20 to 300 °C under a nitrogen atmosphere, with a heating rate of 10 °C/min using the STA 449F3 Jupiter (Netzsch, Selb, Germany) instrument.

Thermogravimetric analysis (TG) was conducted using the following heating program (heating rate: 10 °C/min): heating from 20 °C to 300 °C.

### 2.4. Microstructure Analysis

The cross-section of the fractured composite samples was analyzed using a digital optical microscope Emspira 3 (Leica Microsystems GmbH, Wetzlar, Germany) at specified magnifications as indicated on each micrograph. 

### 2.5. Acoustic Properties Determination

To prepare the natural composite samples, two different methods were used: direct pouring and molding. The direct pouring method involved pouring the heated wax, resin, and filler mixture onto ground cellulose paper pulp in 3D-printed poly (lactic acid) cylindrical molds with 50 mm internal diameter. This method produced filled samples, which were expected to have higher density and lower porosity than the unfilled samples. The molding method involved placing the paper pulp inside cylindrical molds with 50 mm internal diameter, particularly in the interior part composed of a disk with pins with different geometric configurations: circular, undulated, and triangular. The positive pins on the interior disk conferred the desired geometry into the paper core. On the molded pulp, the composite formulation was poured and later extracted after cooling. This method produced samples with different shapes and sizes of voids, which were expected to affect the acoustic properties of the samples. The samples were coded as Pr-x-filled, Pr-x-circ, Pr-x-tri, and Pr-x-und, where “x” denotes the number of the formulation corresponding to Table 1. The use of 3D printing and molding techniques allowed us to create various natural composite samples with controlled and reproducible geometries, which enabled us to investigate the effect of the composition and void shape on the acoustic properties of the samples. 

To evaluate the acoustic properties of the molded composite materials, an acoustic impedance tube (Holmarc HO-ED-A-03, Holmarc Opto-Mechatronics Ltd., Kochi, India) was used. This device consisted of an anodized aluminum tube with an inner diameter of 50 mm, which could measure the sound absorption coefficient (α), the sound transmission loss (TLn), and the reflection coefficient (R) of the samples in the frequency range of 500 Hz–3150 Hz. The device also included two pairs of microphones, sample holders, a data acquisition system, and measurement software. The transfer function method was applied according to the current standards to analyze the frequency dependencies of the acoustic properties of the composite samples. For each sample, several parameters were measured, such as the sample diameter (50 mm), the microphone spacing (30 mm), the temperature, and the humidity. Then, the sample was inserted into the impedance tube between the two sets of microphones in a fixed position. 

## 3. Results and Discussion

In Figures S1–S7 (presented in the Supplementary Materials section), the thermal DSc and TG/DTA thermograms are presented for the beeswax, fir resin, their mixture, and various compositions with ground fir needles, horsetail powder, and rice flour, corresponding to Table 1. Additionally, Figure S8 illustrates the thermograms for the paper pulp. 

The thermal transitions in the negative temp domain and the positive temp domain with low amplitude were not considered in this study for further analysis because they do not affect significant processing or servicing temperatures of natural composites [27,28]. Negative temperature transitions are usually due to the freezing of the traces of bonded and unbonded water within these natural materials [29], and they were not comprehensively considered any further. 

The following aspects were observed from the DSC, DTA, and TG thermograms:

- During stage I (cooling), the crystallization of certain compounds (possibly small molecular) from the studied matrices (beeswax, fir resin) was observed at temperatures of approximately −20 °C. Mixing the two matrices, as well as mixing them with the filler agent (horsetail powder, fir needles, rice flour, recycled paper), leads to a decrease in this crystallization point by approximately 1–3 °C.
- During stage II (heating), beeswax melting was observed at 69.8 °C, and fir resin melting started at 58 °C and continued until 293.5 °C, corresponding to different fractions thereof. Mixtures of the two matrices melt at temperatures 1–2 °C lower than the pure components, and mixing matrices with the filler agent has, essentially, the same effect. Fir resin also exhibits a softening point at 30.4 °C (glass transition temperature, T_g_); the mixture of the two matrices (wax and resin) lowers this point to 15.8 °C. No softening point was detected for pure wax.
- During stage III (cooling), beeswax oxidation begins at 291.4 °C, and fir resin oxidation at 181.3 °C. The mixture of the two matrices (wax and resin) is thermally stable under the analysis conditions, with no exothermic peak corresponding to the oxidation observed. This aspect may be favorable for obtaining composite materials with this type of matrix. The addition of horsetail powder and fir needle powder to wax leads to a slight decrease in the onset temperature of oxidation (288 °C and 285 °C), while in the case of rice flour, the decrease is insignificant (around 1 °C).
- The solidification of melts (wax + fillers, fir resin) always occurs with undercooling, with solidification temperatures lower than those corresponding to melting at stage II.

The data from Figures S1–S8 are summarized in Table 2 for the second and/or third TA regimes, where significant changes occur in the thermograms. Also, the mass loss (expressed in %) is given for the thermal regime end value of 300 °C.

The thermal stability of the composites is influenced by the type and amount of the matrix and the filler materials. Fir resin has a higher thermal stability than beeswax or plant-based materials, as it can withstand higher temperatures without degradation. Therefore, composites with higher fir resin content show higher thermal stability than those with lower fir resin content. Also, fir resin shifts the onsets for oxidation to higher temperatures. Horsetail (Equisetum) is a natural filler that contains a high percentage of inorganic silicate-based compounds, such as silica and phytoliths. These compounds can enhance the thermal stability of the composites by increasing the char formation and reducing the mass loss during thermal decomposition [17,30]. Hence, the composites with horsetail addition exhibit higher thermal stability than those with fir needles or rice flour. 

For comparison, the relevant thermal parameters for two of the most used insulation materials in buildings and the automotive domain, namely, polyurethane foam and textile rags, are also presented in Table 2. The textile rags present the second-highest mass loss evidenced from T_g_, after the composites with rice flour, while the polyurethane foam presented the lowest thermal degradation in the studied temperature interval. The textile rags start to degrade at around 250 °C, about the same as polyurethane, which is 30 °C lower than beeswax and the composites with beeswax matrix, implying that the composites used in this study have the potential to be used in a wider temperature interval. Also, paper pulp presented good thermal stability until 200 °C, which is the onset of oxidation for this material. By adding different natural formulations to paper pulp, its thermal stability can be improved. 

Studying cyclic heating–cooling regimes can help optimize the design and selection of natural composites for various applications that require thermal stability and resistance. By analyzing the thermal properties, such as melting point or onset of oxidation of different natural composites and their combinations under cyclic heating–cooling regimes, the best natural composites and compositions can be identified and improved [31]. The thermal cyclic performance for the matrices and composite mixtures from this study being subjected to five consecutive cooling/heating cycles between −70 °C and 70 °C is illustrated in Table 3. Only the major peaks with the highest enthalpy were selected for each formulation.

Minitab v.19 software was used to compare the peak temperatures for the different compositions and test if the observed changes were statistically significant. The significance level was set at 0.05. The null hypothesis is that there is no difference in the peak temperatures for the different compositions. The alternative hypothesis is that there is a difference in the peak temperatures for the different compositions. In the cases where *p* was higher than 0.05, the differences were statistically significant. For *p* values lower than 0.05, the null hypothesis of no difference can be rejected.

The values presented in Table 3 showed no significant statistical differences in the melting point of all components, except for Pr-9, but in this case, the largest difference could be attributed to the possible elimination of some volatile compounds from beeswax or fir resin, after which the melting point values tend to be stabilized. This is supported by the fact that the *p*-value for Pr-9 was lower than the significance level of 0.05. The values of T_g_ are significantly affected by the cyclic thermal stressing regime, but this is not unusual, taking into consideration that the glass transition temperature is a kinetic parameter, depending more on the heating/cooling regime than the changes in the material. This is consistent with the theory of glass transition, which states that the glass transition temperature is not a fixed property of a material but rather a function of the thermal history and the molecular mobility of the material. The results indicate that the peak temperatures for the different compositions were not significantly affected by the cyclic thermal stress. Therefore, it could be concluded that the studied composites maintained their chemical stability under thermal cycling conditions. While the literature data for all-organic biocomposites’ thermal stressing are missing, there seems to be associated evidence for the thermal stability of plant-based fillers and fibers embedded in synthetic matrices [19,32,33,34,35].

**Figure 1:**
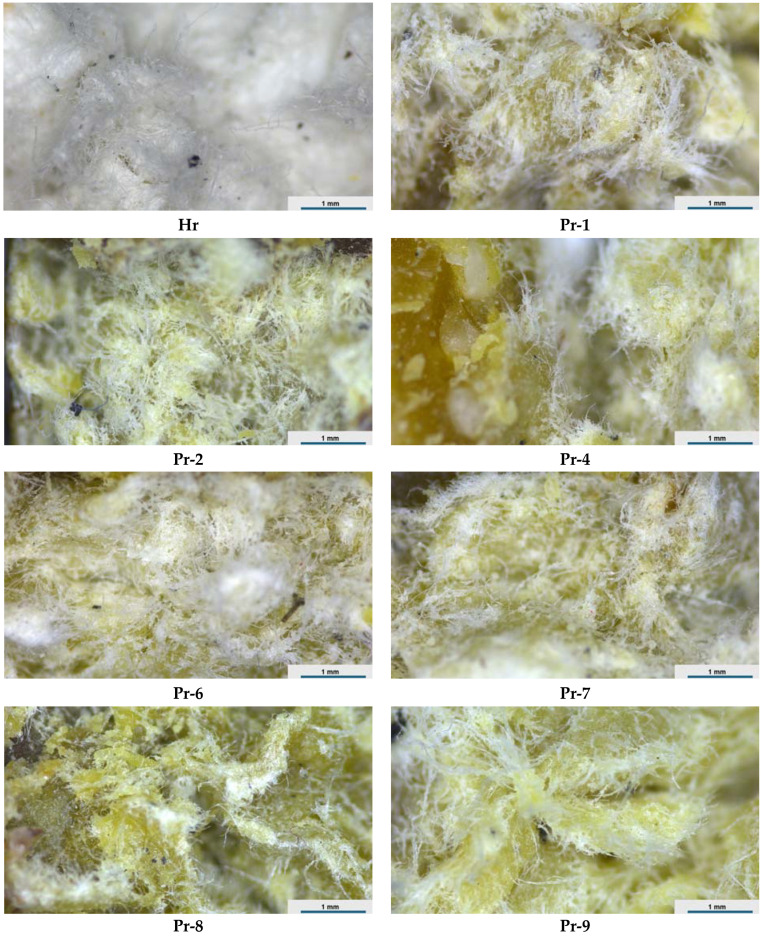
Microscopic images of the obtained composite recipes.

Figure 1 shows the optical microscopy images of the composite samples prepared from different natural materials, as listed in Table 1. The samples were composed of a cellulosic pulp base, onto which a mixture of beeswax, fir resin, and various fillers was added. The fillers included horsetail powder, rice flour, and ground fir needles. Sample Hr (Figure 1) consisted of pure cellulosic pulp microfibers, which had a compact and uniform appearance. The microfibers might also contain some traces of binder, lignin, and other components from the original paper source.

Samples Pr-1 to Pr-9 consisted of cellulosic pulp microfibers embedded with beeswax, fir resin, or their blends, along with different fillers. The integration of horsetail powder, rice flour, or ground fir needles with molten wax or resin imparts unique characteristics to each composite. For instance, samples with horsetail powder exhibit a more granular texture due to the incorporation of fine particles. The uniform distribution of these additives within the cellulosic matrix is crucial for enhancing the mechanical and thermal properties of the composites. The microscopy images illustrate this uniformity and hint at an effective bonding between the cellulosic fibers and added materials. This bonding is essential for achieving enhanced structural integrity and durability. Furthermore, it can be observed that there is a variation in color tones across different samples; this could be attributed to the natural pigments present in horsetail powder, rice flour, or fir needles. These variations not only contribute esthetically but may also influence properties like UV resistance.

An analysis revealed that the wetting of cellulose fibers was optimal with beeswax (samples Pr-1, Pr-4, Pr-6, Pr-7) due to its lower melting viscosity. For fir resin, the results showed the formation of resin-rich clusters near the surface (Pr-2) and that mixing with beeswax improved its ability to penetrate the cellulosic matrix (Pr-8 and Pr-9). Based on microscopic analysis, there is no clear distinction between the ground biomass fillers from the cellulosic fibers, likely due to the homogeneous distribution of the fillers within the cellulose pulp.

One of the objectives of this study was to determine the feasibility of natural composite formulations as sound insulation materials. One feasible hypothesis was that the natural composite formulations would have comparable or better acoustic properties than the conventional sound insulation materials. To test this hypothesis, the sound absorption coefficient and the sound transmission loss of the natural composite samples were measured and compared with the literature values of the conventional materials. In contrast with “filled” materials, the voids and internal patterns of sound-absorbing materials are important factors that affect their acoustic performance, as they influence the interaction of sound waves with the material’s structure. Different shapes and sizes of voids can create different modes of sound absorption, such as viscous dissipation, thermal conduction, and resonance. Different internal patterns can also affect the sound propagation and reflection within the material [36].

The acoustic impedance spectra results for the different compositions formulated in Table 1, as cast on paper pulp and molded with different patterns, are presented below. Three parameters were analyzed from the acoustic impedance spectra, namely the sound transmission loss (TLn), sound absorption coefficient (α), and reflection (R). These parameters quantify the amount of sound energy that is transmitted, absorbed, and reflected by the material, respectively. Sound transmission loss (TLn) is defined as the difference in sound power level between the incident and transmitted sound waves [37]. The sound absorption coefficient (α) is defined as the ratio of the absorbed sound power to the incident sound power [38]. The reflection (R) is defined as the ratio of the reflected sound pressure to the incident sound pressure [39]. These parameters are important for evaluating the acoustic performance of natural composites, as they indicate the effectiveness of the material in reducing noise and improving sound quality.

**Figure 2:**
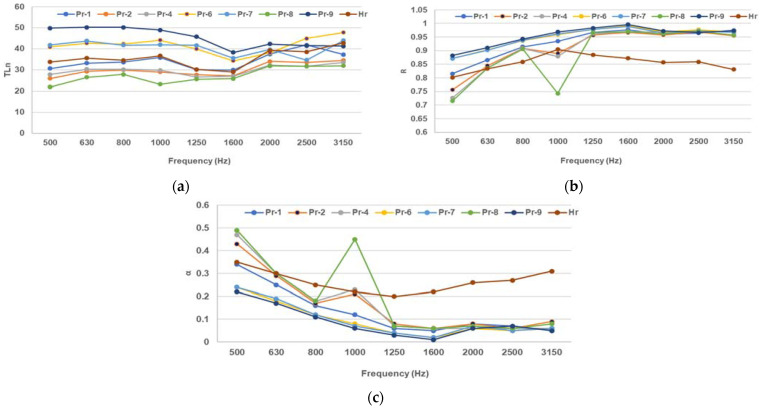
Acoustic measurements for the full molded composites and paper pulp: (**a**) sound transmission loss (TLn), (**b**) sound absorption coefficient, (**c**) sound reflection coefficient.

The results from Figure 2 illustrate the acoustic impedance envelope for the poured and molded composite formulations.

A higher TLn means a lower sound transmission and better sound insulation. All samples show an increase in TLn with frequency, which means they are more effective in blocking high-frequency sound waves (Figure 2a). All the composite samples have higher TLn than Hr, which means they have higher density and lower porosity than paper pulp. Among the Pr samples, Pr-7 (with fir needles and beeswax mixture) has the highest TLn, which means it could have the lowest air gap among the natural composites. The literature values of TLn for porous materials range from 10 dB to 40 dB, depending on the frequency, thickness, and density of the material. The Pr samples have similar or higher TLn than the literature values, which means they have comparable or better sound insulation performance than the usual insulation materials [40].

A higher α means a higher sound absorption and a lower sound reflection. The Pr samples show a peak in α around 1000 Hz, which means they have a resonance frequency at this point (Figure 2b). The resonance frequency depends on the thickness, density, and elasticity of the material. The Pr samples have lower α than Hr (paper pulp) at low and high frequencies, which means they have lower surface area and higher flow resistivity than paper pulp. Among the Pr samples, Pr-1 (paper pulp with beeswax) has the highest α, which means it could have the lowest filler content and the highest air gap among the natural composites. The literature values of α for porous materials range from 0.1 to 0.9, depending on the frequency, porosity, and flow resistivity of the material. The Pr samples have relatively similar or comparable α than the literature values, which means they have comparable sound absorption performance to the usual insulation materials [36].

Conversely, a higher sound reflection coefficient R means a lower sound absorption and a higher sound transmission. The composites show a significant peak in R around 1000 Hz, which corresponds to the resonance frequency of the sound absorption coefficient (Figure 2c). This means that the Pr samples reflect most of the sound energy at this frequency, which reduces their sound absorption performance. The Pr samples have higher R than Hr at most frequencies, which means they have a higher impedance mismatch with the surrounding air than paper pulp. Among the Pr samples, Pr-9 (with fir resin and beeswax mixture) has the highest R, which means it could have the highest density and the lowest porosity among the natural composites. The literature values of R for porous materials range from 0.1 to 0.9, depending on the frequency, impedance, and thickness of the material. The Pr samples have similar or higher R than the literature values, which means they have comparable or higher sound reflection performance than the usual porous materials [36].

An improvement in the acoustic parameters of the material can be obtained by the introduction of patterned voids in the material. This is advantageous due to two main reasons: firstly, the material does not need further modifications (amendments in composition), and second, by introducing gaps, the overall cost or cost per weight parameters present significant improvements.

**Figure 3:**
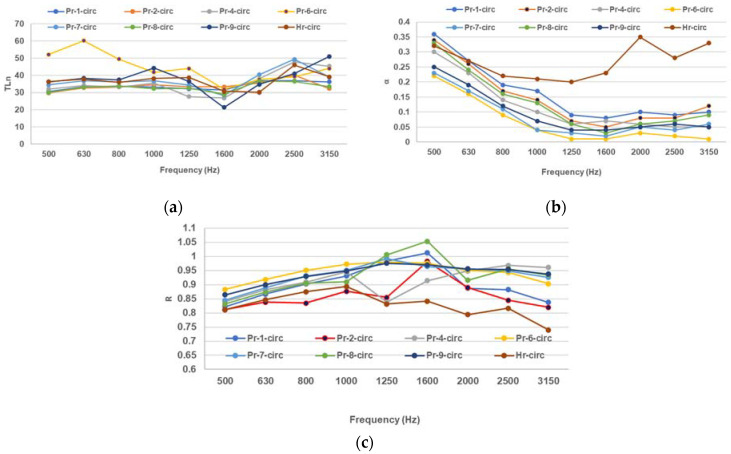
Acoustic measurements for the circular-patterned molded composites and paper pulp: (**a**) sound transmission loss (TLn), (**b**) sound absorption coefficient, (**c**) sound reflection coefficient.

Figure 3 presents the acoustic parameters for the samples obtained with circular patterns.

The composites with circular hole patterns have lower TLn than the filled composites (Figure 3a), which means they have lower density and higher porosity than the filled composites. The composites with circular hole patterns also show more fluctuations in TLn with frequency, which means they have more resonance modes than the filled composites. The composites with circular hole patterns also have higher α than the filled composites at most frequencies, which means they have higher surface areas and lower flow resistivities than the filled composites (Figure 3b). The composites with circular hole patterns have lower R than the filled composites at most frequencies (Figure 3c).

**Figure 4:**
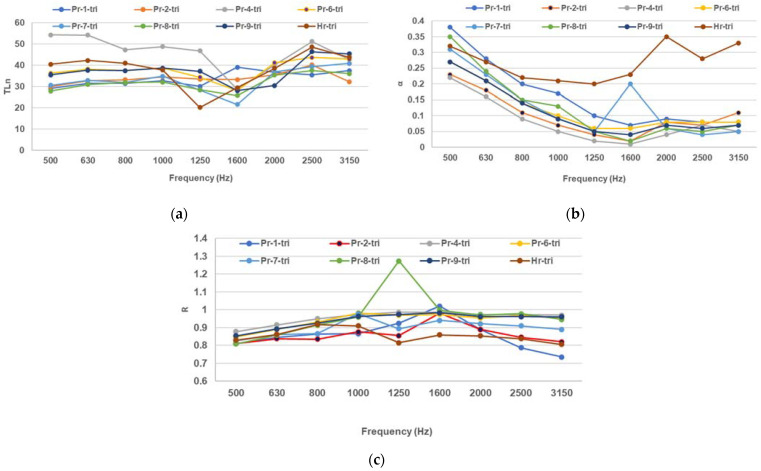
Acoustic measurements for the triangular-patterned molded composites and paper pulp: (**a**) sound transmission loss (TLn), (**b**) sound absorption coefficient, (**c**) sound reflection coefficient.

The composites with triangular pattern voids have lower TLn than the plain and circular-void composites (Figure 4a), higher α than the plain and circular-void composites at most frequencies (Figure 4b), and lower R than the plain and circular-void composites at most frequencies (Figure 4c), which means that there is an improvement in the sound insulative properties and there is a lower number of reflected soundwaves compared to the filled composites and those with circular patterns.

**Figure 5:**
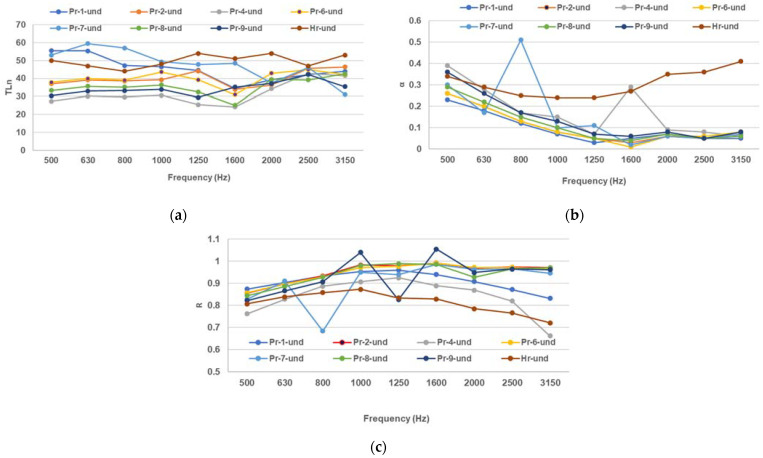
Acoustic measurements for the undulated-patterned molded composites and paper pulp: (**a**) sound transmission loss (TLn), (**b**) sound absorption coefficient, (**c**) sound reflection coefficient.

Among the composites with undulated perforations, the sound transmission loss TLn is, on average, generally lower than the filled composites or the composites with circular or triangular patterns (Figure 5a). Composites with undulated perforations present a higher sound absorption coefficient α than the plain and circular-void composites at most frequencies but lower than the triangular-void ones (Figure 5b).

Complementary to the sound absorption coefficient α, the sound reflection coefficient R presents the lowest values for the undulated pattern composites at most frequencies (Figure 5c).

To summarize the effect of the material type and internal structure on the acoustical parameters, the average values of the acoustic parameters over the whole frequency domain were used, and the top five materials were selected in terms of performance. The average values can reflect the overall acoustic performance of the materials, while the values at a specific frequency may vary depending on the resonance modes and the geometry of the materials. Even if the general trends for the variation in the acoustic parameters were summarized above, compositions from each category of material could present, in some cases, the best performance.

**Figure 6:**
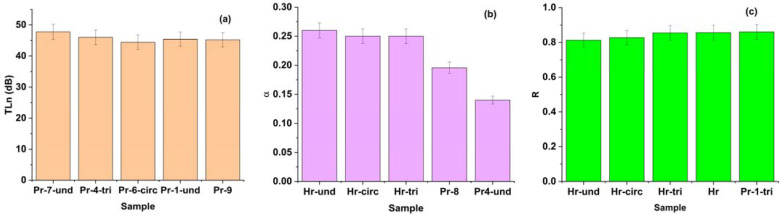
Average values of the selected materials in terms of acoustic performance: (**a**) sound transmission loss (TLn), (**b**) sound absorption coefficient, (**c**) sound reflection coefficient (Hr: paper pulp core; Pr: composites with paper core impregnated with the formulations from Table 1).

The ranking of the composites in terms of the acoustic parameters is presented in Figure 6.

A high TLn means that the material can block most of the sound energy from passing through, which is desirable for sound insulation applications. It can be seen from Figure 6a that the best sound insulation character is registered for sample Pr-7-und, based on a paper core impregnated with beeswax and ground fir needles. However, a high TLn does not necessarily mean a high sound absorption coefficient (α) or a low sound reflection coefficient (R). These parameters measure the amount of sound energy that is absorbed or reflected by the material, respectively. A high α means that the material can reduce the noise level and improve the sound quality, which is desirable for sound absorption applications. A low R means that the material can avoid unwanted echo or noise, which is also desirable for sound absorption applications. Therefore, depending on the application, it may be better to have a high TLn, a high α, or a low R. For example, if the noise from outside sources needs to be reduced, a material with a high TLn is desired. For improvement in the acoustics of a room, a material with a high α and a low R might be more suitable.

The highest sound absorbing properties were registered for the plain paper composites with undulated and circular patterns (Figure 6b), for which the lowest sound reflection coefficient was also registered (Figure 6c). The advantage of using the Pr composites over plain paper is that they have higher density, lower porosity, higher flow resistivity, and higher surface area than plain paper. These factors contribute to the higher sound transmission loss of the Pr composites, which means they have better acoustic performance than plain paper for insulation purposes.

Even if for sound absorption and reflection, the Hr samples (paper pulp) slightly outperformed the composite formulations (Figure 6b,c), the Pr composites still have acceptable acoustic performance for these parameters. The Pr composites have moderate-to-high sound absorption coefficients, ranging from 0.2 to 0.8, depending on the frequency and the pattern. The Pr composites (based on impregnated paper pulp) also have low-to-moderate sound reflection coefficients, ranging from 0.1 to 0.5, depending on the frequency and the pattern. These values are comparable to or slightly lower than the literature values of sound absorption and reflection coefficients for porous materials, such as polyurethane foam, mineral wool, fiberglass, and textile fabrics (Table 4).

In terms of sound transmission loss, the composite formulation with beeswax and fir needles impregnated paper pulp core (Pr-7) and the one with beeswax and horsetail powder impregnated paper pulp core (Pr-4) with undulated or triangular pattern bordered or slightly outperformed the synthetic foams or basalt fiber in terms of their sound transmission loss, which means it can effectively block the sound transmission. Furthermore, as compared to wood-based panels or mineral fibers, paper-core composites have the clear advantage of having higher specific acoustical properties (attributed to their density).

However, the Pr composites also show some drawbacks, such as a significant peak in sound absorption and reflection coefficients around 1000 Hz, which corresponds to the resonance frequency of the materials. This means that the Pr composites absorb and then reflect most of the sound energy at this frequency, which reduces their sound absorption and insulation performance. This peak can be attributed to the thickness, density, and elasticity of the materials, which can be optimized by further modifying the formulation and the pattern of the composites.

The Pr composites, due to their components, could have the advantage of being more durable, stable, and biodegradable than plain paper, which means they have better mechanical, thermal, and environmental properties than plain paper. The Pr composites have higher thermal stability than plain paper, which means they can resist higher temperatures and dissipate heat more efficiently.

Through the simple modification of the obtained technology for the composite panels, namely gravitational pouring (without supplementary pressing of the material in the mold), an improvement in the sound absorption coefficient for the Pr-4 sample (beeswax −62.5% and horsetail powder −37.5%) from 0.15 to 0.58 and the Hr sample from 0.26 to 0.78 can be achieved. The sound absorption behavior is influenced by the micropores, resulting in the composite material when it is not supplementary pressed.

## 4. Conclusions

The utilization of natural materials in sound insulation panels presents a significant advancement in the realm of acoustic engineering, offering a promising alternative to traditional synthetic counterparts. These panels demonstrate exceptional performance across various acoustic parameters, often surpassing conventional materials in specific scenarios. One key advantage lies in their multifaceted composition, incorporating diverse natural components that synergistically enhance their thermal stability, mechanical strength, and environmental sustainability.

The study elucidates the thermal behavior of natural composite materials derived from recycled paper pulp, beeswax, fir resin, and various fillers like horsetail powder, rice flour, and ground fir needles. As evidenced by our findings, these natural composite panels exhibit superior thermal degradation behavior, the onset of degradation being as high as 290 °C compared to polyurethane foams. Also, promising thermal stability under cyclic heating–cooling regimes has been registered, showcasing the potential for applications requiring resistance to thermal stress.

The incorporation of patterned voids in the composites offers a promising avenue for improving their acoustic parameters. Several architectures of these voids were researched in this study, namely circular-, triangular-, and undulated voids, with the undulated ones showing the highest sound absorption and transmission-blocking. Regarding the acoustical dampening, the composites with beeswax and fir needles showed comparable or marginally higher sound transmission loss coefficients to traditional materials such as foams or wood particleboard.

While the results show promise for the use of these biocomposites as insulation or acoustic panels, their suitability is restricted to indoor or climatically stable outdoor applications. Due to the natural organic composition of the matrix and fillers, these materials would likely be vulnerable to degradation if exposed to prolonged cold and damp conditions. However, when encased in shells (made from polymers, polymers composites, wood, metallic, etc.), frames, or in indoor applications, the components are protected from weather factor variations as well as from biological attack by fungi, molds, insects, and rodents.

Future research could focus on further optimizing the composition and structure of these composites to enhance their acoustic performance while maintaining their thermal stability and environmental benefits. By modifying the internal structure and the pressure of molding, the composites can achieve good sound insulation and absorption properties (sound absorption coefficients as high as 0.78, as determined from our preliminary experiments), making them versatile candidates for diverse acoustic applications. Additionally, exploring novel filler materials and refining the manufacturing processes could unlock new possibilities for improving the overall efficiency and applicability of these natural composite materials.

## Figures and Tables

**Table 1:** Material recipes used in this study.

Crt. No.	Material	Sample Code
1	Beeswax (100%)	Pr-1
2	Fir resin (100%)	Pr-2
3	Cellulose pulp (100%)	Hr
4	Beeswax (50%) + fir resin (50%)	Pr-9
5	Beeswax (62.5%) + horsetail powder (37.5%)	Pr-4
6	Beeswax (45.5%) + rice flour (54.5%)	Pr-6
7	Beeswax (61.5%) + ground fir needles (38.5%)	Pr-7
8	Beeswax (31.25%) + fir resin (31.25%) + horsetail powder (37.5%)	Pr-8

**Table 2:** Thermal transitions, oxidation, and mass loss for the analyzed composite mixtures.

Thermal Analysis	DSC/DTA				TG	
Sample Code	TA Stage	Peak Temperature (°C)	ΔH (J/g)	Observations	Mass Loss at 300 °C (%)	Observations
Pr-1	II—heating	68.5	−15.53	Melting	1.27	Highest onset of oxidation
	III—cooling	292.6	3.32	Onset of oxidation		
Pr-2	II—heating	30.4	-	Tg—softening	6.24	
		58.1...293.5	−0.91...−0.97	Melting		
	II—heating	181.3	−0.003	Onset of oxidation		
Pr-9	II—heating	15.8	-	Tg—softening	5.09	Intermediary thermal stability between wax and fir resin
		58.4...291	−3.21	Melting		
	II—heating	-	-	No oxidation was observed		
Pr-4	II—heating	70.6	−26.21	melting	14.60	Highest onset of oxidation from all composites
	III—cooling	288.8	0.29	Onset of oxidation		
	III—cooling	292.6	3.32	Onset of oxidation		
Pr-6	II—heating	71.2	−8.11	Melting	25.53	Lowest thermal stability from all wax matrix composites
		143.3...282.7	−11.09...−0.77	Melting of rice compounds (waxes)		
	III—cooling	290.6	4.83	Onset of oxidation		
Pr-7	II—heating	68.2	−10.89	Melting of wax	14.09	Intermediary thermal stability
		140.3	−4.03	Melting of compounds from fir needles		
	III—cooling	285.9	10.90	Onset of oxidation		
		58.4...291	−3,21	Melting		
Pr-8	II—heating	65.6	−16.87	Melting of wax	7.61	Highest thermal stability from the studied composite mixtures
		174.6...290	−0.35...−0.95	Melting of resin components		
	III—cooling	284.7	0.04	Onset of oxidation		
	III—cooling	284.1	13.95	Onset of oxidation		
Hr	II—heating	73.1	−18.65	Free water vaporisation	20.90	
	III—cooling	−12.2	−0.428	Free water freezing		
	DTA	205.2	−19.7	Onset of oxidation		
Polyurethane foam	II—heating	160.7	0.015	Melting	0.56	
	II—heating	246.2	−0.12	Onset of oxidation		
Textile rags	II—heating	249.8	−4.94	Onset of cellulose oxidation	16.78	

**Table 3:** Cyclic thermal stressing of the natural composite formulations.

Sample Code	DSC Heating Stage Peak	T (°C)					*p* Value at 95% Confidence	Observations
		1st Cycle	2nd Cycle	3rd Cycle	4th Cycle	5th Cycle		
Pr-1	Wax melting	68.5	69.4	68	68.7	68.6	0.92	No significant changes
Pr-2	Resin softening point (T_g_)	30	33.4	38.1	43.8	44.1	0.00049	Significant changes
	Resin melting	58.1	60	58.4	58.5	58.6	0.97	No significant changes
Pr-9	softening point (T_g_)	15.8	22.6	21.7	38.4	48.5	0.00015	Significant changes
	Melting	58.4	66.2	65.3	65.2	65.3	0.0064	Significant changes
Pr-4	Melting	70.6	69.6	68.5	68.3	68.3	1	No significant changes
Pr-6	Melting	71.2	70	69.3	68.8	68.7	1	No significant changes
Pr-7	Melting	68.2	71.5	69.5	69.4	69.4	1	No significant changes
Pr-8	Melting	65.6	64	63.2	63.5	63.5	1	No significant changes

**Table 4:** Acoustical performance of traditional insulators from literature data.

Material	Density (kg/m^3^)	Thickness (mm)	Sound Absorption Coefficient (α)	Sound Transmission Loss (dB)	Reflection Coefficient (R)	References
Mineral wool	30–200	25–200	0.8–1.0	40–60	0.2–0.4	[41]
Recycled basalt fibers with bio-binders	10–100	10–50	0.6–0.9	20–40	0.3–0.6	[42]
Polyurethane foams	20–80	10–50	0.6–0.9	20–50	0.3–0.6	[43]
Polystyrene foams	10–40	10–50	0.2–0.4	10–30	0.7–0.9	[44]
Wood particleboard composites	300–800	10–20	0.4–0.7	15–35	0.5–0.8	[45]
Recycled textile materials	10–100	10–50	0.4–0.8	15–30	0.4–0.8	[46]

## Data Availability

Data are contained within the article. Supporting information can be made available on request by the corresponding author.

## Supplementary Materials

The following supporting information can be downloaded at: https://www.mdpi.com/article/10.3390/polym16050672/s1, Figure S1: (a): DSC and (b): TG/DTA thermograms for beeswax (Pr-1). Figure S2: (a): DSC and (b): TG/DTA thermograms for fir resin (Pr-2). Figure S3: (a): DSC and (b): TG/DTA thermograms for Beeswax (50%) + fir resin (50%) (Pr-9). Figure S4: (a): DSC and (b): TG/DTA thermograms for Beeswax (62.5%) + horsetail powder (37.5%) (Pr-4). Figure S5: (a): DSC and (b): TG/DTA thermograms for Beeswax (45.5%) + rice flour (54.5%) (Pr-6). Figure S6: (a): DSC and (b): TG/DTA thermograms for Beeswax (61.5%) + ground fir needles (38.5%) (Pr-7). Figure S7: (a): DSC and (b): TG/DTA thermograms for Beeswax (31.25%) + fir resin (31.25%) + horsetail powder (37.5%) (Pr-8). Figure S8: (a): DSC and (b): TG/DTA thermograms for the paper pulp sample (Hr).
