# Supplementary material for: Thermal and Sound Insulation Properties of Organic Biocomposite Mixtures
Source: Polymers (Basel). 2024 Mar 1;16(5):672. doi: 10.3390/polym16050672 (PMC10934450; doi:10.3390/polym16050672)
Supplement: Supplementary file 1 [file polymers-16-00672-s001.zip › polymers-2890189-supplementary.pdf]

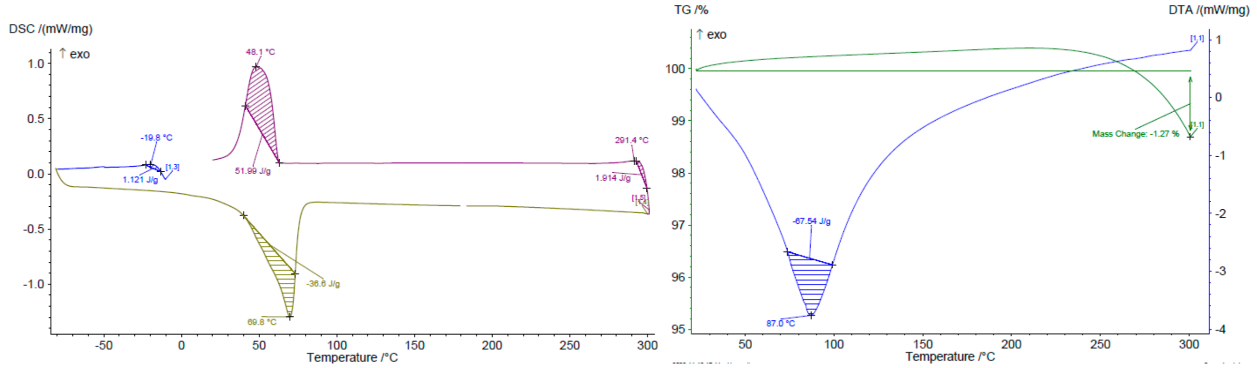

(a) **(b)**

Figure S1. (a): DSC and (b): TG/DTA thermograms for beeswax (Pr-1).

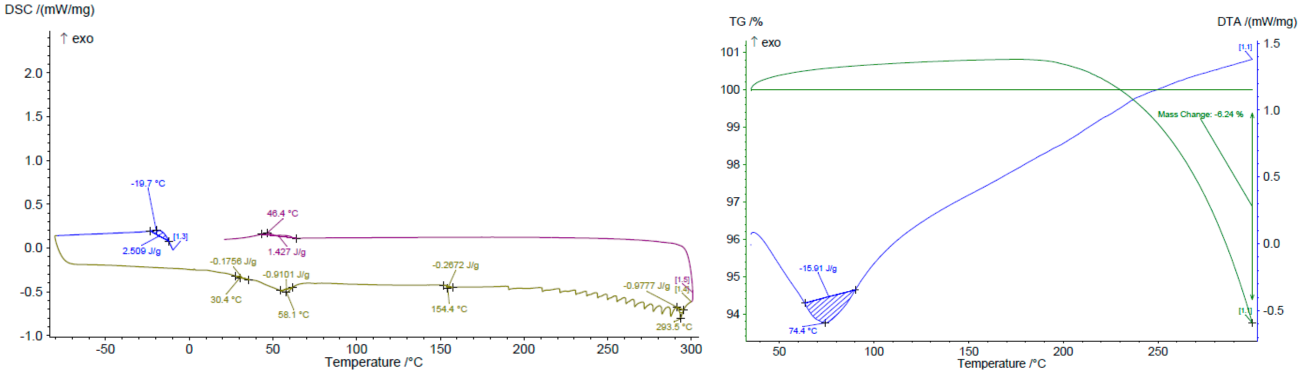

(a) **(b)**

Figure S2. (a): DSC and (b): TG/DTA thermograms for fir resin (Pr-2).

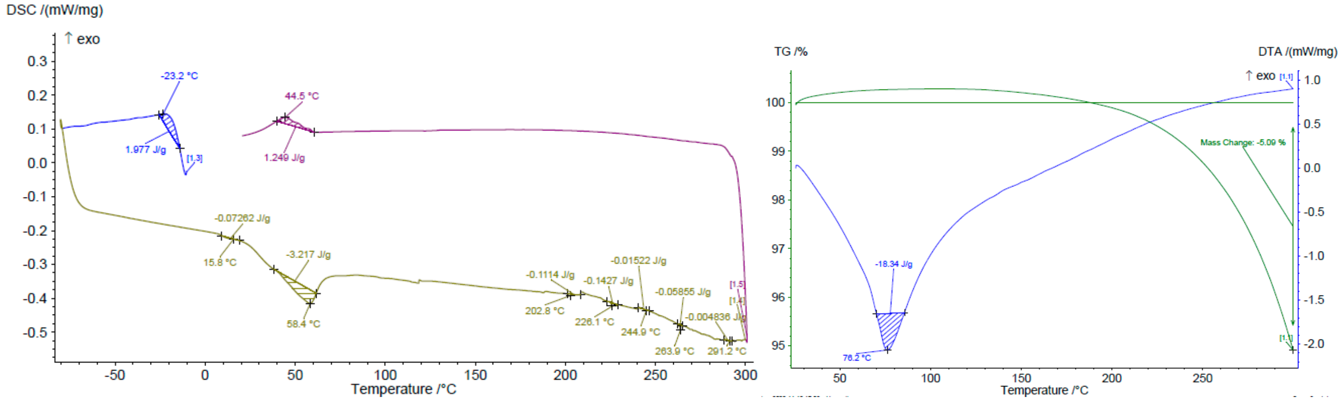

(a) **(b)**

Figure S3. (a): DSC and (b): TG/DTA thermograms for Beeswax (50%) + fir resin (50%) (Pr-9).

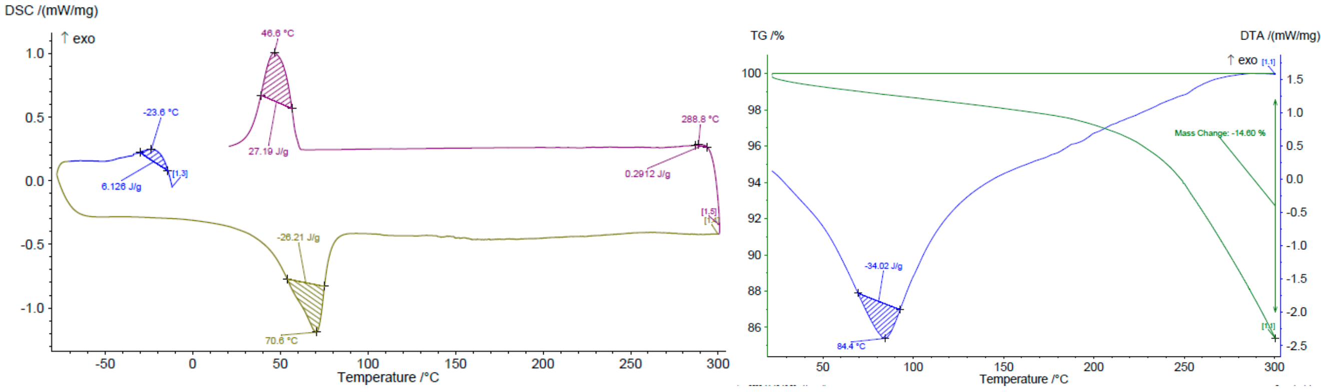

(a) **(b)**

Figure S4. (a): DSC and (b): TG/DTA thermograms for Beeswax (62.5%) + horsetail powder (37.5%) (Pr-4).

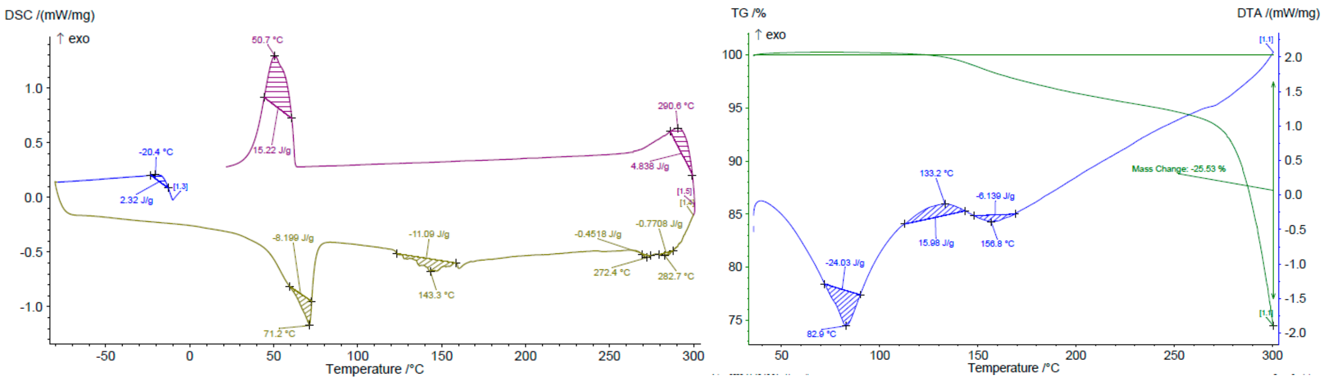

Figure S5. (a): DSC and (b): TG/DTA thermograms for Beeswax (45.5%) + rice flour (54.5%) (Pr-6).

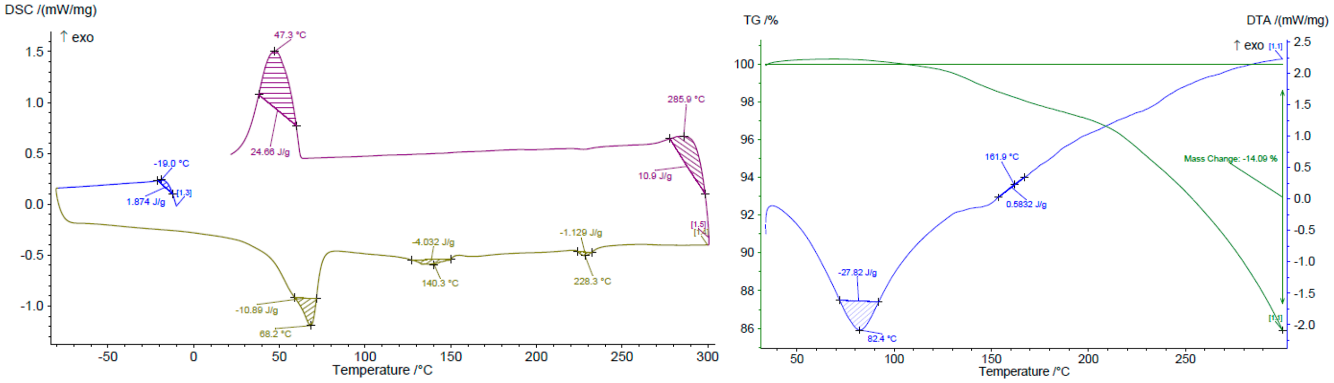

Figure S6. (a): DSC and (b): TG/DTA thermograms for Beeswax (61.5%) + ground fir needles (38.5%) (Pr-7).

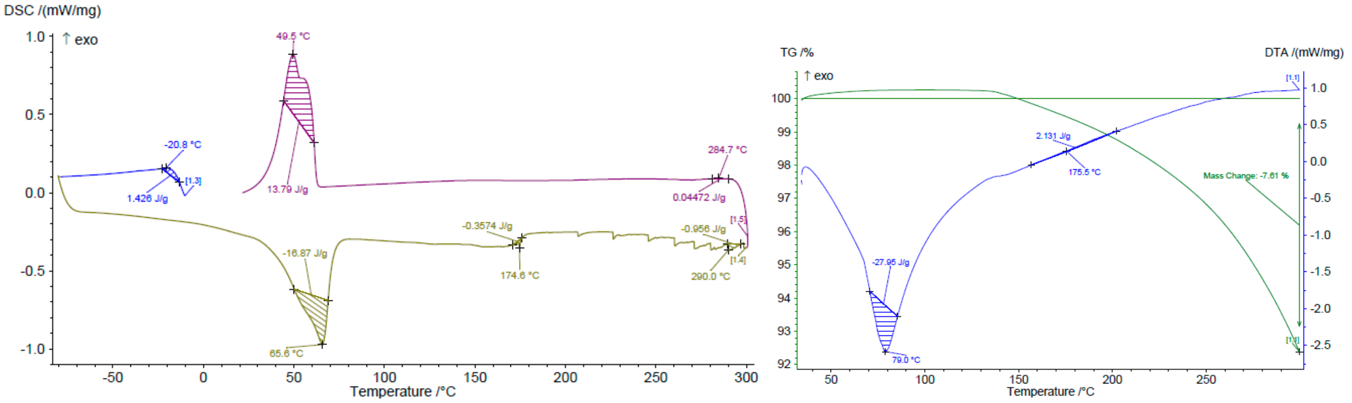

Figure S7. (a): DSC and (b): TG/DTA thermograms for Beeswax (31.25%) + fir resin (31.25%) + horsetail powder (37.5%) (Pr-8).

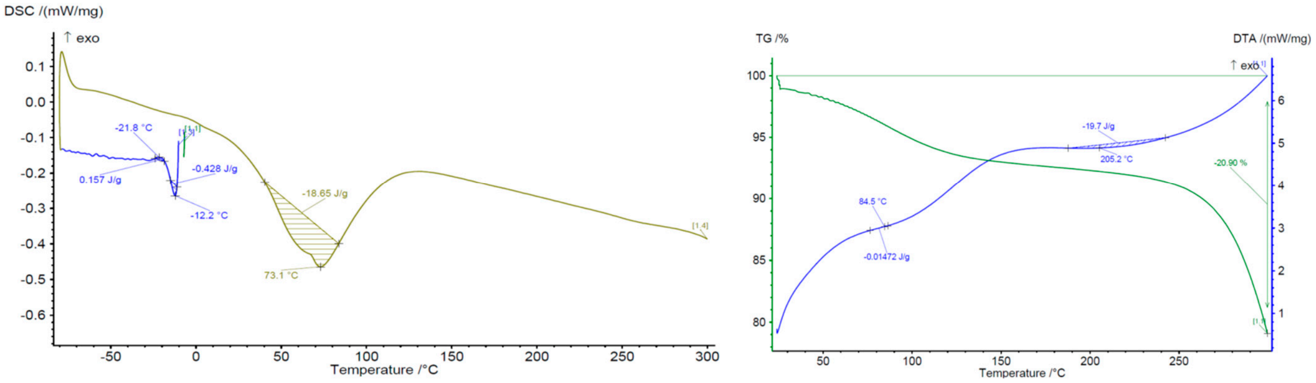

Figure S8. (a): DSC and (b): TG/DTA thermograms for the paper pulp sample (Hr).
